# Infectivity of adeno-associated virus serotypes in mouse testis

**DOI:** 10.1186/s12896-018-0479-1

**Published:** 2018-11-01

**Authors:** Santhanasabapathy Rajasekaran, Jayashree Thatte, Jayaprakash Periasamy, Alok Javali, Manjunath Jayaram, Dwaipayan Sen, Akshaya Krishnagopal, Giridhara R. Jayandharan, Ramkumar Sambasivan

**Affiliations:** 1 Institute for Stem Cell Biology and Regenerative Medicine, GKVK Campus, Bellary Road, Bengaluru, 560065 India; 2 National Centre for Biological Sciences, TIFR, GKVK Campus, Bellary Road, Bengaluru, 560065 India; 3 Department of Haematology and Centre for Stem Cell Research, Christian Medical College, Vellore, 632004 India; 4 Cellular and Molecular Therapeutics Laboratory, Centre for Biomaterials, Cellular and Molecular Theranostics (CBCMT), Vellore Institute of Technology (VIT), Vellore, 632014 India; 5 Department of Biological Sciences and Bioengineering, Indian Institute of Technology, Kanpur, 208016 India

**Keywords:** Adeno-associated viruses (AAV), Germline transmission, Tropism, Mouse testis injection, Myoid cell, Leydig cell, Sertoli cell, Spermatogonia

## Abstract

**Background:**

Recombinant adeno-associated viruses (AAVs) are emerging as favoured transgene delivery vectors for both research applications and gene therapy. In this context, a thorough investigation of the potential of various AAV serotypes to transduce specific cell types is valuable. Here, we rigorously tested the infectivity of a number of AAV serotypes in murine testis by direct testicular injection.

**Results:**

We report the tropism of serotypes AAV2, 5, 8, 9 and AAVrh10 in mouse testis. We reveal unique infectivity of AAV2 and AAV9, which preferentially target intertubular testosterone-producing Leydig cells. Remarkably, AAV2 TM, a mutant for capsid designed to increase transduction, displayed a dramatic alteration in tropism; it infiltrated seminiferous tubules unlike wildtype AAV2 and transduced Sertoli cells. However, none of the AAVs tested infected spermatogonial cells.

**Conclusions:**

In spite of direct testicular injection, none of the tested AAVs appeared to infect sperm progenitors as assayed by reporter expression. This lends support to the current view that AAVs are safe gene-therapy vehicles. However, testing the presence of rAAV genomic DNA in germ cells is necessary to assess the risk of individual serotypes.

**Electronic supplementary material:**

The online version of this article (10.1186/s12896-018-0479-1) contains supplementary material, which is available to authorized users.

## Background

Adeno-associated viruses (AAVs) are promising gene therapy vectors as AAV-mediated gene delivery is very efficient and safe [1]. However, germ line transmission of the transgene delivered by AAVs is a safety concern in the field [2, 3]. On the other hand, AAVs with limited genome integration [4] could serve as potent tools to deliver transgenes for generating animal models. Here, transplantation of germ cells engineered in vitro with AAV has been promising [5, 6]. An easier alternative is direct testicular injections of AAVs to target male germ line. In either scenario, gene therapy or transgenesis in animal model, it is crucial to know the infectivity of the AAV serotypes in testis.

AAV are non-enveloped viruses of *parvoviridae* family with a single-stranded DNA genome of 4.7 kilobases (kb), packaged in a capsid protein of icosahedral symmetry. AAVs possess the ability to infect both dividing and non-dividing cells [7]. Different serotypes of AAVs are known to have tropism towards different cell types [8]. The basis of tropism specificity is the polymorphism of capsid protein. Knowledge of infectivity of different serotypes within a given tissue or organ is valuable in gene therapy context.

The stem cells of the male germ line spermatogonial stem cells (SSCs) and their descendant spermatogonial cells are located within the seminiferous tubules. The tubules are formed by epithelial Sertoli cells. Spermatids produced from spermatogonia reach lumen of the tubules. The tubules are barricaded by an epithelial layer of peritubular contractile myoid cells. The myoid cell epithelia along with the Sertoli cell epithelia form the formidable blood-testis barrier in rodents [9, 10]. Outside the myoid cell barrier, testosterone-producing Leydig cells and blood vessels occupy the interstitial niche among the tubules. Testicular injection introduces the AAVs in the interstitial space exterior to the myoid cell layer. There is scant information on the infectivity of different AAV serotypes in testis.

Here, we report infectivity of a number of AAV serotypes within testis upon injection in mouse testis capsule. Except two, all serotypes tested efficiently target interstitial cells. Specifically, AAV2 and AAV9 uniquely transduced Leydig cells. Notably, a phosphomutant of AAV2 serotype engineered to improve virion survival, displayed a dramatically altered tropism. It traversed myoid cell barrier and infected Sertoli cells, but did not transduce Leydig cells. In spite of direct injection into testis at moderate to high titre, none of the tested serotypes infect SSCs. Thus, our findings support their label as safe vehicles for gene therapy.

## Results

### Wild type AAVs preferentially target Leydig cells

**Fig. 1:**
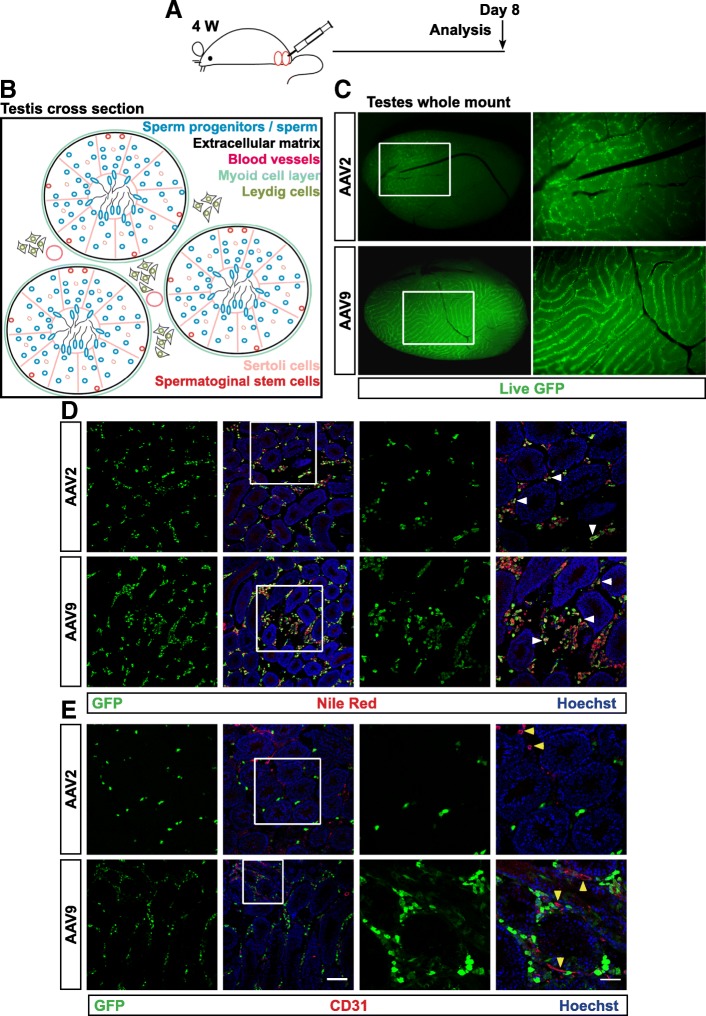
AAV serotypes tested primarily target Leydig cells. **a** Schematic of the experiment. Direct testicular injection of EGFP encoding viral suspension into the intertubular space in 4 weeks old C57/BL6:DBA2 F1 hybrid males. Viral particles were injected in the right testis of each animal and the left testis served as uninjected control; *n* = 3 animals. **b** Illustration of a mouse testis cross-section. **c** Wholemount of dissected testis showing distribution of live GFP from infected cells. **d** Combined immunostaining for GFP and Nile red stain (white arrowheads) reveal transduction of Leydig cells by AAV2 and AAV9. **e** Cryosections immunostained with endothelial marker CD31. Yellow arrowheads highlight absence of GFP and CD31 co-expression. Scale bar 50 and 100 μm, for low and high magnification images, respectively

**Fig. 2:**
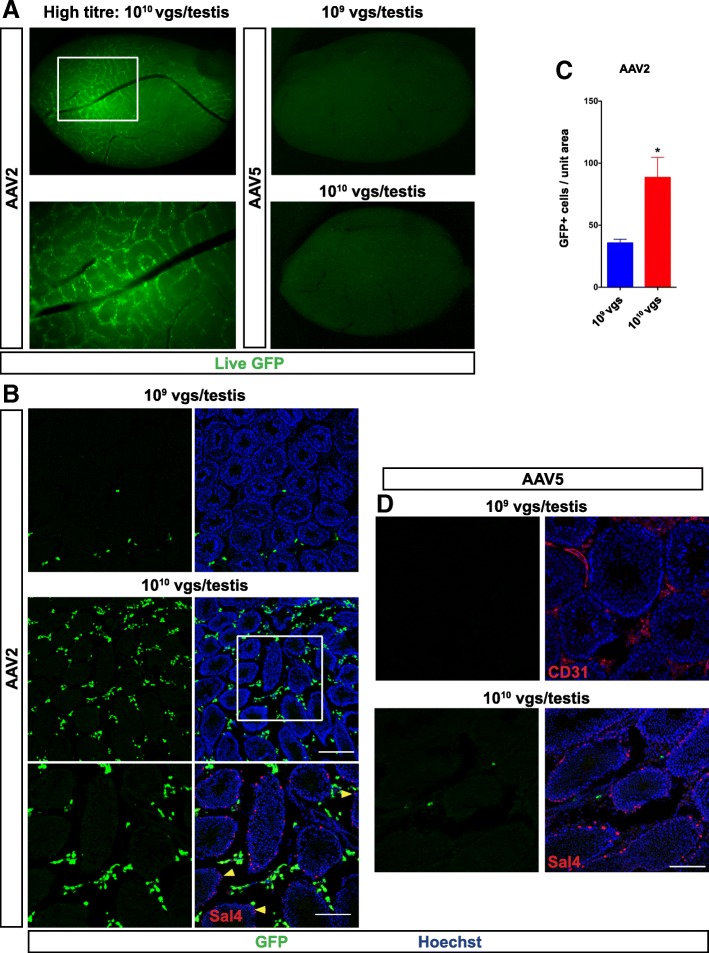
Infection at higher titre does not alter distribution. Higher titre of AAV2 and AAV5 at 1 X 10^10^ viral genomes (vgs) were injected per testis and analysed 1 month post-injection, *n* = 3 animals. **a** Wholemount of dissected testis showing distribution of live GFP. **b** Cryosections immunostained with anti-GFP antibody to detect AAV2 transduced cells. Note the distribution of transduced cells outside the seminiferous tubules; SSCs marked by Sal4 are not transduced (Yellow arrowheads). **c** Graph showing increase in transduction efficiency with higher titre. The number GFP+ cells / 0.58 mm^2^ (mean ± standard deviation; 52.78 ± 16.45; *p < 0.01; n = 3 animals). **d** Cryosections immunostained with anti-GFP antibody to detect AAV5 transduced cells. Rare transduced cells were observed with AAV5 injected at 1 X 10^10^ vgs per testis. Scale bar 50 and 100 μm, for low and high magnification images, respectively

To investigate the tropism of AAV serotypes in testis and infectivity of sperm progenitors, we injected AAVs of different serotypes into the interstitial space of the mouse testis (Fig. 1a, b; schematics of the experiment, testis cross section). Since, the Sertoli cell mediated blood-testis barrier develops at puberty, we injected 4 weeks old prepubescent animals to test possible viral distribution in the adluminal compartment of seminiferous tubules. Our thymidine analog 5-ethynyl-2′-deoxyuridine (EdU) incorporation assays showed that a large number of sperm progenitors are in the proliferative compartment during this period (Additional file 1: Figure S1A). We tested five different serotypes AAV2, 5, 8, 9 and AAVrh10 at 1 X 10^9^ AAV viral genomes (vgs) per testis (see Methods). All serotypes have enhanced green fluorescent protein (EGFP) expression cassette flanked by AAV2 inverted terminal repeats, but pseudo-typed with capsid proteins of the different serotypes. Majority of the serotypes have been reported to show expression at the site of injection within a week of injection [8]. Therefore, we analyzed bio-distribution of all five serotypes 8 days following injection. Widespread transduction was observed in testes injected with AAV2, 9 and 10 by live GFP expression on whole mount, while AAV5 and 8 showed no or few transduced cells, respectively (Fig. 1c; Additional file 1: Figure S1B). To assess AAV distribution in testis, immunofluorescence was performed on testes cryosections for the virally encoded GFP. For AAV2, 9 and 10, our analysis revealed GFP+ transduced cells uniquely in the intertubular spaces after 8 days (Fig. 1d; Additional file 1: Figure S1B). To quantitate the transduction efficiency we enumerated GFP+ cells on cryosections. In accordance with the wholemount GFP expression, AAV2, 9 and 10 showed higher efficiency compared to AAV 5 and 8 (Additional file 1: Figure S1C; see Fig. 2c for AAV2). Next, we addressed the specific cell type transduced in the testis. Staining with lipophilic Nile red showed that testosterone-producing Leydig cells, which contain large lipid droplets are targeted by AAV2 and AAV9 (Fig. 1d). However, endothelial cells of the vasculature immunostained with CD31, also in the intertubular space, are not targeted by AAV2 or AAV9 (Fig. 1e). Thus, it appears that the unique target population of AAVs, at least of AAV2 and AAV9 serotypes, are Leydig cells outside the seminiferous tubules and that they do not infect tubules or intratubular cells.

The myoid cell layer around the tubules is a potential barrier that could shield tubules from AAVs. In fact, the peritubular myoid cells circumscribe the developing seminiferous tubules and begin to express Collagen 1 in foetal testis [11, 12]. While, Collagen 1 component of the extracellular matrix (ECM) derives exclusively from myoid cells, Laminin is secreted by Sertoli cells [13]. We have performed AAV injections at 4 weeks. By this stage, the myoid cell epithelial barrier and ECM are fully formed as shown by comparison of 3, 4 and 8 weeks old testes using smooth muscle actin-α (myoid cell layer), Collagen 1 and Laminin 5 (ECM markers) as well as integrin β1 (apical membrane of Sertoli cells; Additional file 2: Figure S2). Thus, it is likely that AAVs fail to traverse the peritubular myoid cell or the ECM barrier.

To test the impact of increasing the viral titer on distribution within testis, we injected AAV2 at 1 X 10^10^ AAV viral genomes (vgs) per testis and analyzed 8 days post injection. At this higher titer, AAV2 showed similar transduction pattern of GFP+ cells uniquely in the interstitium (Fig. 2a, b). Sal4+ SSCs outlining the periphery of the tubules clearly showed that increasing the titer did not result in viral infiltration across myoid cell barrier nor transduction of SSCs (Fig. 2b). However, quantitation of GFP+ cells revealed more efficient transduction with higher titer (Fig. 2c). Higher titer AAV5 injection (10^10^ vgs per testis) resulted in rare GFP+ cells in interstitial space (Fig. 2d). Thus, most wildtype AAV serotypes tested infect intertubular cell types and did not infiltrate across the myoid layer.

### Engineered AAV2 mutant displays novel tropism in testis

**Fig. 3:**
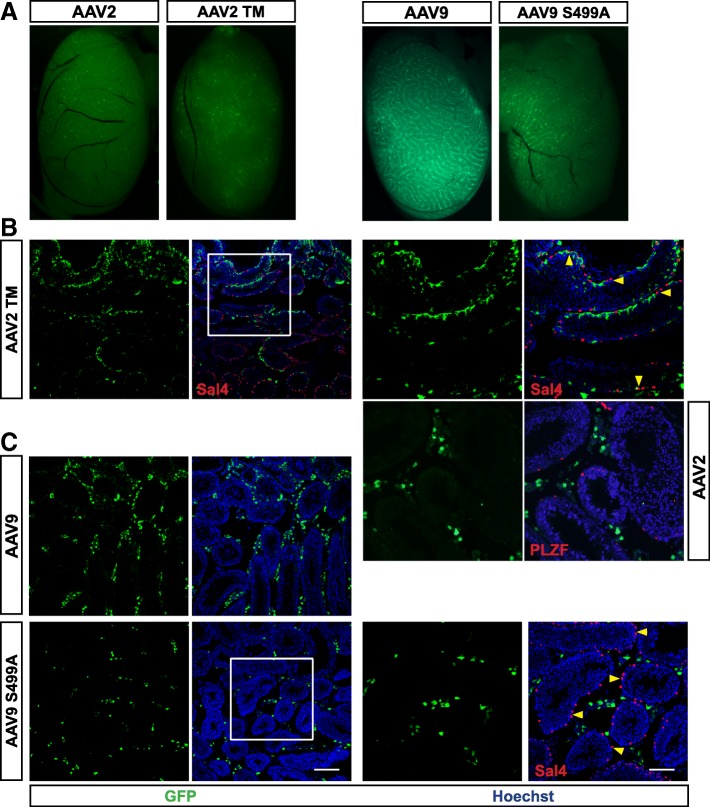
Tropism of Phosphodegron mutants of AAV2 and AAV9. AAV2 TM (triple mutant carrying S489A, T251A and K532R mutations) and AAV9 S499A were injected into 4 weeks old males. 1 X 10^9^ vgs / testis; n = 3 animals. **a** Testes wholemount showing live GFP expression. **b** Confocal microscopic analysis comparing wildtype AAV2 with that of AAV2 TM. Sal4 and PLZF are spermatogonial stem cell markers. The images show the distribution of GFP+ cells in the seminiferous tubules in AAV2 TM injected testis. Yellow arrowheads highlight Sal4+ SSCs, which are GFP negative. **c** Immunostained cryosections of testis comparing wildtype AAV9 with that of AAV9 S499A mutant transduction (See Fig. S1C for quantitation). Scale bar 50 and 100 μm, for low and high magnification images, respectively

**Fig. 4:**
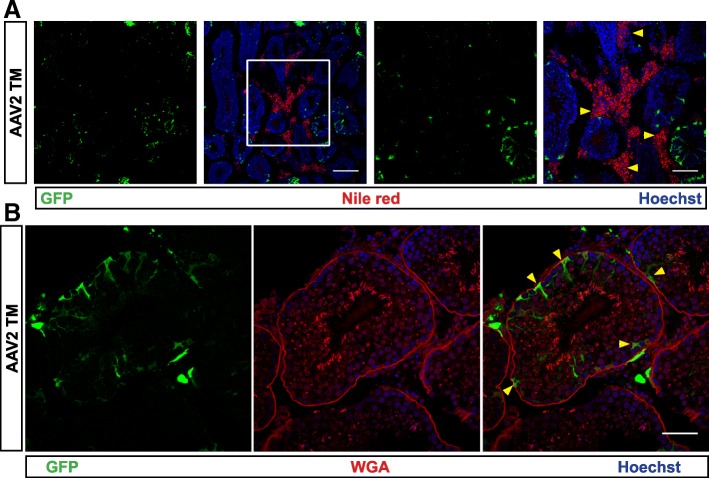
Phosphodegron mutant AAV2 TM displays novel tropism. **a** Confocal microscopic analysis of testis cryosections stained with lipophilic Nile red and immunostained for GFP. Nile red positive Leydig cells (yellow arrowheads) are not transduced by AAV2 TM. Scale bar 50 and 100 μm, for low and high magnification images, respectively. **b** Wheat germ agglutinin staining (WGA) staining combined with GFP immunostaining. Pattern of cytoplasmic GFP (yellow arrowheads) indicate transduction of Sertoli cells; WGA high spermatogonial derivatives are GFP negative. Scale bar 50 μm

The viral particles are targeted for ubiquitin-mediated proteasome degradation machinery by phosphorylation of specific residues on the capsid. Mutations of these phosphodegrons improve the transduction efficiency of AAV2 as well as other serotypes [14–16]. In parallel to testing wildtype serotypes, we had tested one mutant each of AAV2 and AAV9 for infectivity in testis. AAV2 TM is a triple mutant with residue changes S489A, T251A and K532R, while AAV9 mutant was an S499A modification described previously [17, 18]. The mutants used here, showed improved transduction on cultured HeLa cells or in hepatic gene transfer in mice over their wildtype counterparts [17, unpublished data]. The mutant serotypes were injected in the testes of 4 weeks old males and analyzed 1 month later. AAV9 S499A mutant is less efficient in transducing cells in murine testis compared to its cognate wildtype serotype (Fig. 3a, c; Additional file 1: Figure S1C). Remarkably, we found GFP+ cells in the tubules upon testicular injection of AAV2 TM (Fig. 3a, b). Moreover, Nile red staining showed that AAV2 TM did not infect Leydig cells (Fig. 4a). To determine the cell type infected by AAV2 TM within the seminiferous tubules, cryosections were stained with a membrane marker, wheat germ agglutinin (WGA) that binds to N-acetyl-D-glucosamine on plasma membrane and has been extensively used to identify spermatogenic cells [19]. Confocal imaging showed GFP expression in the epithelial Sertoli cells with the typical branched morphology, extending from the basement membrane to the lumen of the tubules (Fig. 4b). However, no GFP expression was observed in sperm progenitors at different stages of differentiation, which are brightly marked by WGA (Fig. 4b). Thus, we conclude that AAV2 TM primarily target Sertoli cells and not SSCs or its derivatives. Overall, the results indicate a fully altered tropism for AAV2 within testis upon mutation of the residues chosen to improve virion infectivity.

## Discussion

Evaluating the infectivity of AAV serotypes in various organs and tissues is key to exploit this nucleic acid delivery vehicle for gene therapies. Assessing preferential tropism of AAVs in testis is key to asses safety of AAVs from the perspective of vertical gene transmission. In contrast, serotypes with a germ line targeting potential will be valuable for achieving stable transgenesis in animal models using CRISPR (clustered regularly interspaced short palindromic repeats) technology. Our data attest to the safety of AAVs as gene therapy vehicles. However, we show striking alteration in tropism in testis upon engineering AAV capsids for improving transduction. Thus, our report emphasizes the need for reassessment of tropism of engineered AAVs to ensure they are safe for gene therapies.

Our data represents a thorough analysis of distribution of a set of AAVs when injected directly in the testis capsule. This mode of delivery allows more rigorous evaluation of infectivity within testis than systemic delivery. We show that AAV2 and AAV9 efficiently transduce testosterone-producing Leydig cells. Endothelia of blood vessels occupying the same interstitial space as Leydig cells are not transduced revealing specificity of AAV2 and AAV9 for the testosterone producing cells. None of the tested wildtype AAVs appeared to infiltrate into seminiferous tubules or infect sperm progenitors. It is possible that the CAG chimeric promoter-enhancer sequence used to drive GFP reporter is not expressed in the sperm progenitors. However, a recent work has shown that this enhancer is active in sperm progenitors [20]. Remarkably, this study shows transduction of SSCs and downstream sperm progenitors by AAV1 and AAV9 [20]. We speculate that the higher titres used by this study compared to that in our experiments may explain the apparent conflict in the results between the two studies. Moreover, the dilution of episomal rAAV vector genomes in proliferating sperm progenitors may have further reduced the possibility of detecting AAV9 transduction of SSCs in our study.

Differential tropism of AAV serotypes arises due to capsid protein polymorphism [8]. We show that specific mutations of putative phosphodegrons on AAV2 capsid confers novel infectivity. Notably, the triple mutant AAV2 crosses myoid barrier and infects Sertoli cells but does not transduce Leydig cells revealing a complete switch in tropism within testis compared to its cognate wildtype. Thus, our observations suggest that it is possible to engineer AAVs to target SSCs for transgenesis in animal models. In fact, rational design based on knowledge of ligand-receptor interactions mediating virus entry, as well as high-throughput screening using capsid peptide-display combined with selection by directed evolution, both have been successful approaches to engineer AAVs of desired tropism [21, 22]. On the other hand, the finding that mutations aimed to increase the transduction by averting proteasome-mediated degradation significantly changes infectivity of a specific AAV serotype underscores the importance of screening altered tropism while designing AAVs with increased stability or increased transduction efficiency.

Although AAV2 serotype is considered a safe gene therapy vector with respect to vertical transmission, it is documented to transduce murine spermatognia-derived cells in culture [6]. This infectivity may be due to the removal of myoid cell barrier and / or due to the culture-induced changes in spermatogonia-derived cells, because, our study clearly shows that wild type AAV2 does not transduce them in vivo. In rodents, myoid cells form a single layer with tight junctions and this epithelium-like layer is a component of the blood-testis barrier [9, 10]. Therefore, it is likely that many wildtype AAVs do not efficiently cross the peritubular myoid barrier and thus, attest to their safety in the context of gene therapy.

## Conclusions

The alteration in tropism in a capsid mutant designed to improve transduction highlights the necessity of rigorous assessment of tropism of engineered AAVs. Importantly, our results provide a strong support for existing literature and affirm safety of AAV gene therapy vectors owing to its low germ line transmission potential. Nevertheless, we have not assayed for the presence of rAAV genomic DNA in germ cells. This is important for future studies aimed at assessing the risk of germ line transmission of individual serotypes.

## Methods

### Animals

Animals were sourced from the mouse facility at the Institute of Stem Cell Biology and Regenerative Medicine (InStem). F1 hybrids of C57BL/6 J and DBA2J were used for experiments. These inbred strains were originally sourced from The Jackson Laboratory, USA and subsequently, maintained as inbred colonies at the InStem facility. Animals were euthanized by CO2 inhalation as per the institutional guidelines.

### Viral vectors

The AAV helper plasmids were from Agilent technologies (Stratagene, Santa Clara, CA, USA) and the AAV packaging plasmids were a kind gift from Dr. Arun Srivastava, University of Florida, Gainesville. Highly purified stocks of self-complementary wild-type (WT) AAVs or the mutant AAV vectors encoding the enhanced green fluorescent protein (EGFP) gene driven by the chicken β-actin promoter containing the CMV enhancer and SV40 poly A signal were generated by polyethyleneimine based triple transfection of AAV-293 cells (Stratagene). Briefly, 40 dishes (150mm^2^) of 80% confluent AAV 293 cells were transfected with AAV rep-cap plasmid, transgene containing plasmid and AAV-helper free (p.helper) plasmid. Cells were collected 72 h post transfection, lysed and treated with 25 units/ml of benzonase nuclease (Sigma Aldrich, St Louis, MO, USA). Subsequently, the vectors were purified by iodixanol gradient ultra-centrifugation [23] (Optiprep, Sigma Aldrich) followed by column chromatography (HiTrap Q column, GE Healthcare, Pittsburgh, PA). The vectors were finally concentrated to a final volume of 0.5 ml in phosphate buffered saline (PBS) using Amicon Ultra 10 K centrifugal filters (Millipore, Bedford, MA). The physical particle titres of the vectors were quantified independently thrice by slot blot analysis and the mean value was expressed as viral genomes (vgs)/ml [24]. Site-directed mutagenesis was performed to generate AAV2 Triple mutant vector containing the S489A, T251A and K532R mutations and a single mutant AAV9 S499A [17, 18] using QuikChange II XL Site-Directed Mutagenesis Kit (Stratagene, La Jolla, CA, USA) following the manufacturer’s protocol. Briefly, a one-step PCR amplification of the target sites was performed for 18 cycles with the primers (sequence available on request) followed by *DpnI* digestion for 1 h. 2 μL of this digested PCR product was then transformed into XL10-Gold Ultracompetent Cells (Stratagene). Following plasmid isolation, the presence of the desired point mutation was verified by DNA sequencing (Applied Biosystems 3130 Genetic Analyzer, Warrington, UK).

### Testicular injections

Male mice, F1 hybrids of C57BL/6 J and DBA2J were used for experiments and injections performed as previously reported [25]. The animals were anesthetized by Isoflurane (2-chloro-2-(difluoromethoxy)-1,1,1-trifluoro-ethane), surgical site was sterilized with ethanol and topical application of Betadine. A single incision was made on the ventral skin and body wall about 1.5 cm anterior to the genitals, using sterile surgical scissors under aseptic conditions. The testes were pulled from the scrotal sac holding the fat pad. The volumes of the viral stocks were adjusted with PBS to achieve either 1 X 10^9^ vgs or 1 X 10^10^ in 15 μl volume. Each testis was injected 15 μl of the viral suspension using 30G needle syringe. The typical titre we obtain in AAV preparations in laboratory scale is 10^11^–10^12^ viral genomes / ml and the upper limit of the injection volume in the mouse testis capsule is 15 μl. Injection was into intertubular spaces, also known as testis capsule. In a set of animals, left testis served as an uninjected control. The animals were sacrificed after desired period of incubation (3 or 8 days or 4 weeks) post injection and testes were dissected for analysis.

### Immunostaining and fluorescence microscopy

Testes were dissected and imaged wholemount for GFP expression using Stereo zoom microscope Leica M205FA and Leica DFC 3000G monochrome camera. For immunostaining analysis, testes were fixed in 4% PFA in PBS for 45 min at 4 °C and thoroughly washed with PBS. The samples were embedded in Tissue-Tek O.C.T. compound and snap cryofrozen. Cryosections of 10–16 μm thickness were taken on Superfrost plus slides and analysed by fluorescence immunostaining. Random transverse sections were chosen for analysis. For PLZF antibody staining, antigen retrieval was done, wherein the sections were boiled in a solution of 10 mM sodium citrate, pH 6.5 for 3 min. For all staining, blocking and permeabilization was performed in PBS containing 10% FBS and 0.5% TritonX100. Then, sections were immunostained overnight with primary antibody. Antibodies used were: chick- GFP (Abcam; ab13970, 1/1000), PLZF (PG Pelicci, IFOM, Italy, 1/700), Collagen I (Abcam; ab21286, 1/250), smooth muscle actin-α (Abcam; ab137734, 1/200), integrin β1 (Abcam; ab95623, 1/150). The sections were then washed and incubated with secondary antibodies (Donkey raised Alexa-Fluor antibodies from Molecular probes, 1/500). For Nile red staining (Sigma; 7248), following secondary antibody sections were incubated with 0.5 μg/ml Nile red in PBS for 10 min at room temperature. For wheat germ agglutinin staining (WGA), sections were incubated with WGA in PBS for 1 h, the tissues were post-fixed with 4% paraformaldehyde for 15 min and then, incubated with primary and secondary antibody. EdU was injected intraperitoneally at a concentration of 200 μg / g body weight of animals (Injection volume 100 μl). Four EdU pulses were administered in 48 h (12 h interval), animals were sacrificed and the dissected testes were cryosectioned for analysis. EdU incorporation was analysed using Click-iT® EdU Alexa Fluor® 488 Imaging Kit as per the manufacturer’s instruction (ThermoFisher Scientific) following immunostaining. The slides were mounted in 75% Tris-buffered glycerol and images were acquired using Olympus FV100 confocal microscope and Olympus IX73 or BX53 fitted with Olympus DP26 or DP72 camera using CellSens software.

### Quantitation and statistical analysis

For each testis, the number of GFP+ cells per 10X field (0.58 mm^2^) was averaged across 3 fields. Mean value from *n* = 3 animals is represented with standard deviation. Statistical analysis was performed using GraphPad Prism 5.0 Software. Unpaired t test with Welch’s correction was performed at 5% level of significance (*p* < 0.05) to assess significance of observed difference in the infectivity between moderate and high titre serotypes.

## Additional files

Additional file 1: **Figure S1.** Distribution of transduction efficiency of AAVs in mouse testis. A) Immunostaining with EdU staining. Edu incorporation and staining in 4 to 5 weeks old males reveals proliferating sperm progenitors in the seminiferous tubules outlined by Laminin 5 staining. B) Top: Wholemount of dissected testis imaged for live GFP. Bottom: Cryosection immunostained for GFP. Scale bars 100 μm. C) Histogram representing the transduction efficiency of the various serotypes. For each testis, the number of GFP+ cells in a 10X field was enumerated; the number GFP+ cells / 0.58 mm^2^ (mean ± standard deviation; AAV5, 0; AAV8, 26 ± 14.1; AAV9, 104 ± 24.1; AAV9 S499A, 45.3 ± 10.4; AAVrh10, 78 ± 15.1; *n* = 3 animals; *n* = 2 animals for AAV 8). (PDF 2291 kb)

Additional file 2: **Figure S2.** Immunostained cross sections of testis at 3-, 4- and 8- week-old adult testis. α-Smooth muscle actin (α-Sma) is a myoid cell marker. Collagen 1 and Laminin 5 mark the ECM and β1 integrin marks the periphery of seminiferous tubules. Scale bar 50 μm. For 3 right panels of the 4 week time point, scale bar 100 μm. (PDF 6157 kb)

## Abbreviations

(E)GFP: (enhanced) green fluorescent protein
AAV: Adeno-associated viruses
ECM: Extra cellular matrix
EdU: 5-ethynyl-2′-deoxyuridine
SSC: Spermatogonial stem cell
WGA: Wheat germ agglutinin CRISPR Clustered regularly interspersed short palindromic repeats
