# Supplementary figures and images for: Infectivity of adeno-associated virus serotypes in mouse testis
Source: BMC Biotechnol. 2018 Nov 1;18:70. doi: 10.1186/s12896-018-0479-1 (PMC6211462; doi:10.1186/s12896-018-0479-1)

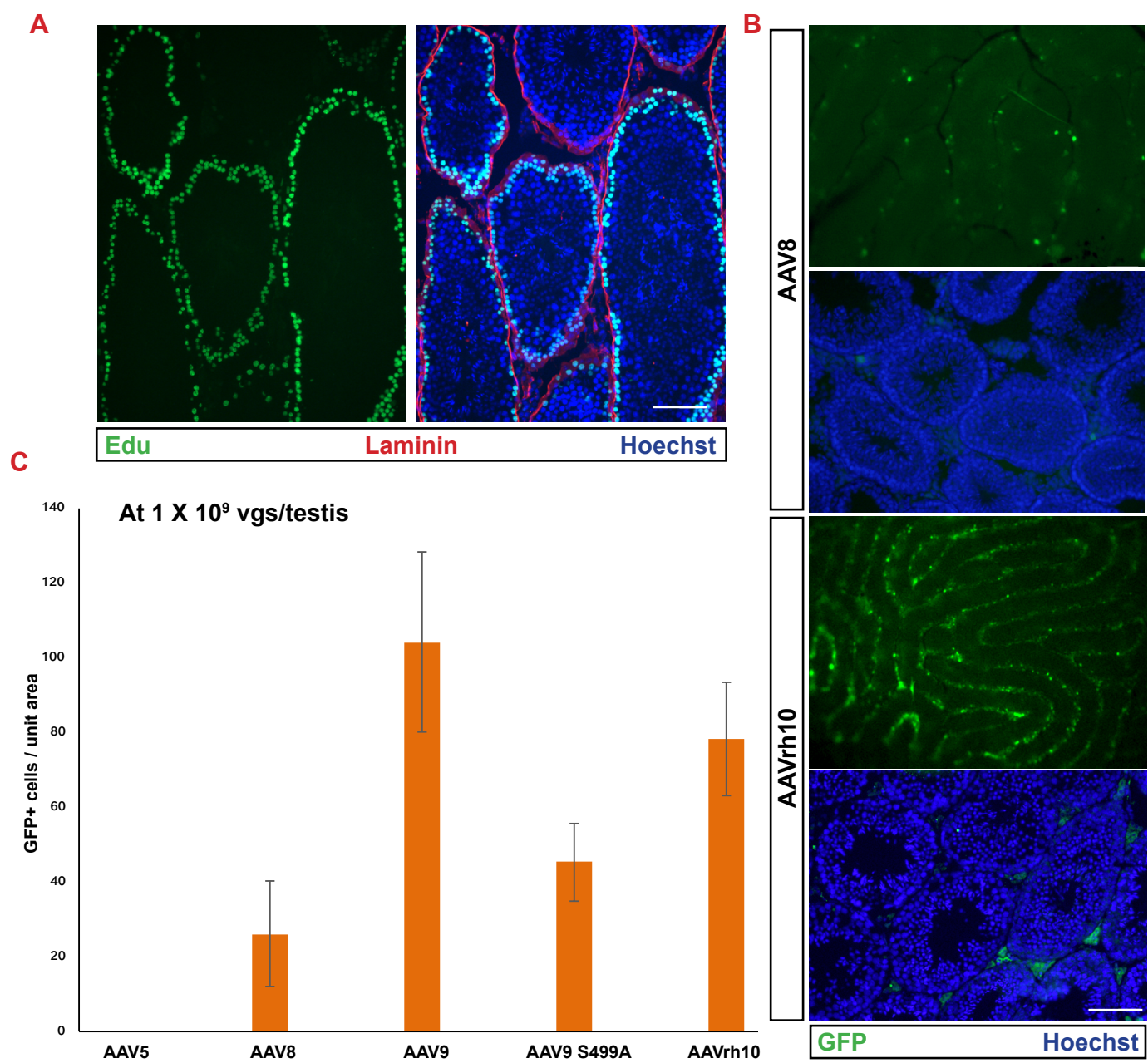

Figure S1

Supplement: Supplementary file 1 — Figure S1. Distribution of transduction efficiency of AAVs in mouse testis. A) Immunostaining with EdU staining. Edu incorporation and staining in 4 to 5 weeks old males reveals proliferating sperm progenitors in the seminiferous tubules outlined by Laminin 5 staining. B) Top: Wholemount of dissected testis imaged for live GFP. Bottom: Cryosection immunostained for GFP. Scale bars 100 μm. C) Histogram representing the transduction efficiency of the various serotypes. For each testis, the number of GFP+ cells in a 10X field was enumerated; the number GFP+ cells / 0.58 mm2 (mean ± standard deviation; AAV5, 0; AAV8, 26 ± 14.1; AAV9, 104 ± 24.1; AAV9 S499A, 45.3 ± 10.4; AAVrh10, 78 ± 15.1; n = 3 animals; n = 2 animals for AAV 8). (PDF 2291 kb) [file 12896_2018_479_MOESM1_ESM.pdf]

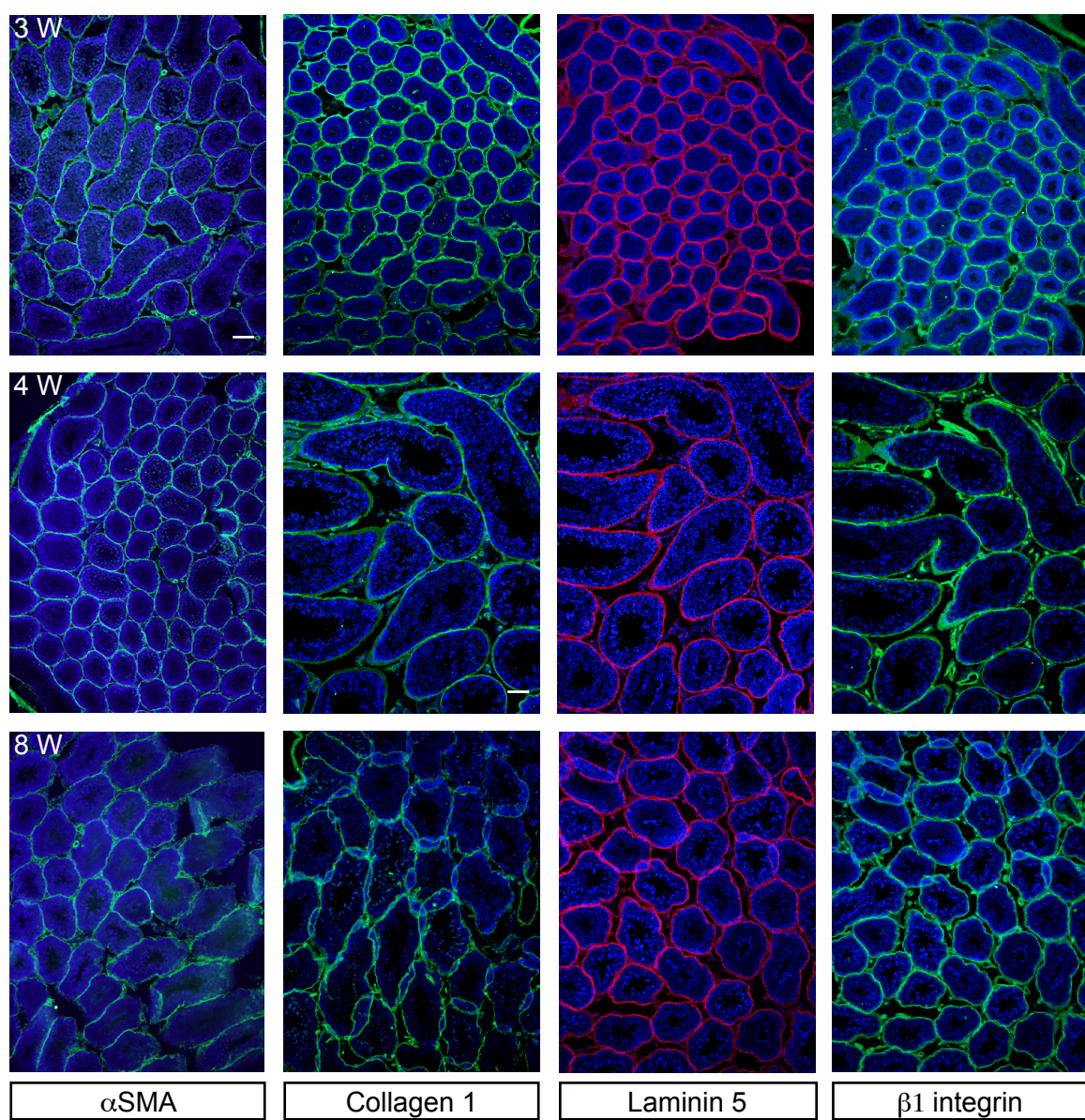

**Figure S2**

Supplement: Supplementary file 2 — Figure S2. Immunostained cross sections of testis at 3-, 4- and 8- week-old adult testis. α-Smooth muscle actin (α-Sma) is a myoid cell marker. Collagen 1 and Laminin 5 mark the ECM and β1 integrin marks the periphery of seminiferous tubules. Scale bar 50 μm. For 3 right panels of the 4 week time point, scale bar 100 μm. (PDF 6157 kb) [file 12896_2018_479_MOESM2_ESM.pdf]
